# The saw-tooth sign as a clinical clue for intrathoracic central airway obstruction

**DOI:** 10.1186/1756-0500-5-388

**Published:** 2012-07-29

**Authors:** Akira Nakajima, Takeshi Saraya, Saori Takata, Haruyuki Ishii, Yoko Nakazato, Hidefumi Takei, Hajime Takizawa, Hajime Goto

**Affiliations:** 1 Departments of Respiratory Medicine, Kyorin University School of Medicine, 6-20-2 Shinkawa, Mitaka City, Tokyo, 181-8611, Japan; 2 Departments of General Surgery, Kyorin University School of Medicine, Tokyo, 181-8611, Japan

**Keywords:** Saw-tooth sign, Obstructive sleep apnea syndrome, Intrathoracic central airway obstruction, Lung cancer, Three-dimensional thoracic computed tomography, Flow volume curve

## Abstract

**Background:**

The saw-tooth sign was first described by Sanders et al in patients with obstructive sleep apnea syndrome as one cause of extrathoracic central airway obstruction. The mechanism of the saw-tooth sign has not been conclusively clarified. The sign has also been described in various extrathoracic central airway diseases, such as in burn victims with thermal injury to the upper airways, Parkinson’s disease, tracheobronchomalacia, laryngeal dyskinesia, and pedunculated tumors of the upper airway.

**Case presentation:**

A 61-year-old man was referred to our hospital with a two-month history of persistent dry cough and dyspnea. He was diagnosed with lung cancer located in an intrathoracic central airway, which was accompanied by the saw-tooth sign on flow-volume loops. This peculiar sign repeatedly improved and deteriorated, in accordance with the waxing and waning of central airway stenosis by anti-cancer treatments.

**Conclusion:**

This report suggests that the so-called saw-tooth sign may be found even in intrathoracic central airway obstruction due to lung cancer.

## Background

The saw-tooth sign was first described by Sanders et al.[1] patients with *obstructive* sleep apnea syndrome. Until today, various diseases found to be associated with this sign. However, no report has been found the sign occurring in patients with lung cancer. We described here the first case of the saw-tooth sign in a patient with lung cancer, which showing the sign could be seen even in an intrathoracic central airway stenosis, depending on its severity during the phase of anti-cancer treatment.

## Case presentation

**Figure 1:**
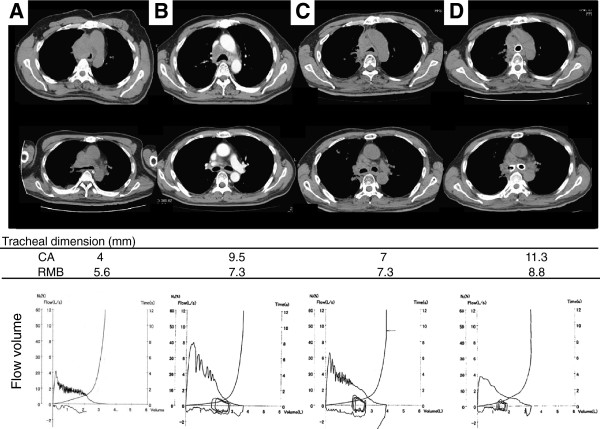
**Sequential findings on thoracic CT and flow-volume loops.** Sequential findings on thoracic CT and flow-volume loops from the day of admission (day1) to the day224. At day1 (Figure 1-A) showed a severe narrowing of the trachea,to approximately 4mm in diameter with moderate stenosis (5mm) in the right main bronchus, which accompanied by saw-tooth sign with trapezoidal shape on flow-volume loops. At day60 (Figure 1-B) revealed the improvement of the limitation of expiratory flow with the disappearance of the saw-tooth sign or trapezoidal shape as well as the amelioration of the stenosis both in the central airway and right main bronchus. At day207 (Figure 1-C), flow-volume loops showed the reproduction of the saw-tooth sign together with the progression of stenosis in the central airway (7mm), but not in the right bronchus. At day224 (Figure 1-D), after completion of inserting stent, saw-tooth sign completely disappeared.

**Figure 2:**
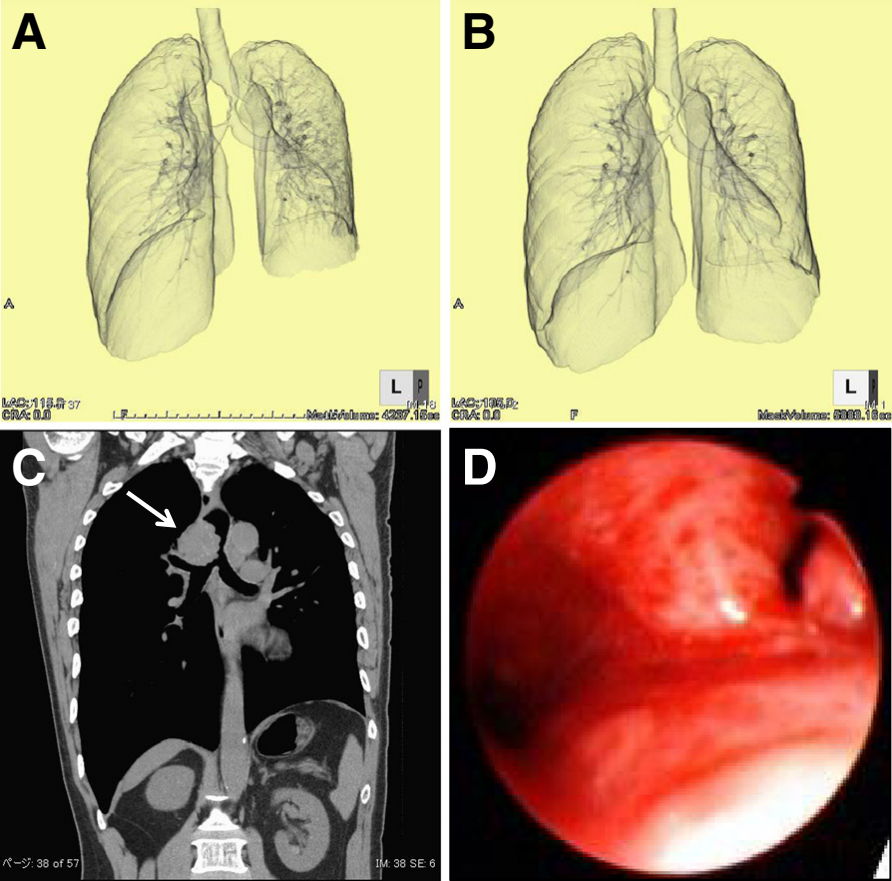
**Multidisciplinary assessment of tracheal stenosis located in intrathoracic area.** Three-dimensional CT at day1 both in the deep expiratory (Figure 2-A) and inspiratory phases (Figure 2-B) clearly depicts the airtrapping only in the deep expiratory phase (Figure 2-A) in the right hemithorax compared with the left hemithorax. Coronal image of thoracic CT on the same day (Figure 2-C) demonstrates the 4-cm mass located along the trachea to the right main bronchus, which severely compresses the intrathoracic upper airway, resulting in narrowing to a 4-mm-diameter. Bronchoscopy performed on the day of admission after intubation (Figure 2-D) shows the protruding tumor in the right main bronchus with mucosal edema, which occupies the almost entire tracheal lumen.

A 61-year-old man was referred to our hospital because of a two-month history of persistent dry cough and dyspnea. He had a myocardial infarction three years earlier. He worked as a programmer. His vital signs, physical examination, and serum laboratory examinations were normal. Non-enhanced thoracic CT on admission (day 1) showed the tumor in the intrathoracic central airway (Figure 1-A), which showed a severe narrowing of the trachea, to a diameter of approximately 4 mm. Interestingly, further evaluation by three-dimensional (3D) CT taken both on deep expiratory (Figure 2-A) and inspiratory phases (Figure 2-B) revealed marked air trapping on the deep expiratory phase in the right lung (Figure 2-A) compared with that in the left lung, accompanied by protrusion of the intrathoracic tumor into the right main bronchus (Figure 2-C, arrow). Bronchoscopy performed on Day 2 confirmed the stenosis of the right main bronchus with mucosal edema by the tumor (Figure 2-D), but no definite evidence of an obstructive cause was noted in the extrathoracic area.

Thereafter, based on the pathological findings of the specimens obtained by transbronchial biopsy, he was diagnosed with lung squamous cell carcinoma (clinical stage of T4N3M0, stage IIIb). Then, 60 days after commencing treatment with cisplatin (80 mg/m^2^) and irinotecan (60 mg/m^2^) followed by radiation therapy (60 Gy/20 fractions/4 weeks), he was found to have a partial response, with improvement in the limitation of expiratory flow, based on the forced expiratory volume in 1 second (Figure 1-A, B). The tracheal and bronchial dimensions measured on thoracic CT showed improvement both in the central airway (CA) and in the right main bronchus (RMB) from Day 1 (Figure 1-A, CA 4 mm, RMB 5.6 mm) to Day 60 (Figure 1-B, CA 9.5 mm, RMB 7.3 mm), respectively. Interestingly, after completion of 4 cycles of chemotherapy, thoracic CT taken on Day 207 showed re-growth of the tumor in the central airway but not in the right main bronchus, together with reproduction of the saw-tooth sign (Figure 1-C, CA 7 mm, RMB 7.3 mm). On Day 224, insertion of a stent was performed for progressive central airway stenosis, which resulted in the disappearance of the saw-tooth sign and amelioration of stenosis, especially in the CA (Figure 1-D, CA,11.3 mm; RMB, 8.8 mm). After inserting the stent, his AHI (apnea hypopnea index) score on polysomnography showed a slightly increased value of 7, but the mean SaO2 was above 92% throughout. In addition, there were no clinical findings of OSAS.

## Discussion

The saw-tooth sign was first described by Sanders et al. [1] in patients with obstructive sleep apnea syndrome (OSAS) and was defined as three or more consecutive peaks and troughs occurring at regular intervals of no greater than 300 cc. The present case satisfied the criteria, with a trapezoidal shape (Figure 1-A), which suggested upper airway obstruction. Sanders et al [1] found that the saw-tooth sign was highly sensitive (85%) and specific (100%) for OSAS, while, after the first report, the sign has also been described in burn victims with thermal injury to the upper airways, Parkinson’s disease, tracheobronchomalacia, laryngeal dyskinesia [2-5], and pedunculated squamous cell carcinoma arising in the pharynx [6]. Thus, based on the recent reports, this sign might not be specific for OSAS. The upper airway is roughly classified into extrathoracic and intrathoracic portions, and the saw-tooth sign has been believed to be a hallmark of OSAS, a representative pattern of extrathoracic obstruction, whereas the present case had an intrathoracic obstruction, and there was no evidence of OSAS or Parkinson’s disease, both clinically and/or polysomnographically. Furthermore, thoracic CT taken on Day 1 (Figure 1, Figure 2-A, B, C) showed stenosis only in the intrathoracic area but not in the extrathoracic area. The inspiratory flow volume curve failed to delineate the whole picture, which had the potential for overlooking the functional site of narrowing. However, based on the above clinical findings obtained by thoracic CT and bronchoscopy, the saw-tooth sign in the present case was considered to have been generated by the obstruction in the intrathoracic area.

Although the mechanism of the saw-tooth sign has not been conclusively defined, this phenomenon has been ascribed to turbulent flow due to intermittent narrowing of the upper airway created by fluttering tissue. However, the present case did not show fluttering tissue on bronchoscopy. Verbraecken et al. [7] reported the pathophysiological aspects of sleep-disordered breathing with a focus on upper airway mechanics in obstructive or central sleep apnea, Cheyne-Stokes respiration, and obesity hypoventilation syndrome. They stated that increased upper airway collapsibility is one of the mechanisms of sleep-disordered breathing, which might be applied in the present case. In addition, the saw-tooth sign might also be associated with bronchoscopically visible edema, which could have increased airway compliance and functioned as an oscillator in the upper airway without fluttering tissue in the present case.

The present case is the first report showing a process generating the saw-tooth sign in a patient with lung cancer, and the sign could be seen even in an intrathoracic central airway stenosis, as shown in a case with tracheobronchomalacia [8].

## Conclusion

This report highlights the saw-tooth sign, which tell us that we should consider the possibility of structural and/or functional upper airway obstruction, either extrathoracic (i.e., OSAS) or involving an intrathoracic central airway when it is evident.

## Consent

Written informed consent was obtained from the patient for publication of this case report and any accompanying images. A copy of the written consent is available for review by the Editor-in-Chief of the journal.

## Competing interests

The authors declare that they have no competing interests.

## Authors’ contributions

AN, TS, ST, and HI managed the patient as primary physicians. AN and TS were primarily responsible for preparing the manuscript. HT inserted the stent for tracheal stenosis. HT and HG had responsibility for all aspects of management as chiefs of our respiratory department. All authors read and approved the final manuscript.

## References

[B1] Sanders MH Martin RJ Pennock BE The detection of sleep apnea in the awake patient. The 'saw-tooth' sign. JAMA 1981 245 2414 2418 10.1001/jama.1981.03310480030021 7230472

[B2] Haponik EF Munster AM Wise RA Upper airway function in burn patients. Correlation of flow-volume curves and nasopharyngoscopy Am Rev Respir Dis 1984 129 251 257 6696326

[B3] Schiffman PL A "saw-tooth" pattern in Parkinson's disease Chest 1985 87 124 126 10.1378/chest.87.1.124 3965257

[B4] Vincken W Cosio MG "Saw-tooth" pattern in the flow-volume loop Chest 1985 88 480 481 4028864 10.1378/chest.88.3.480-a

[B5] Newhouse MT Martin L Kay JM Laser resection of a pedunculated tracheal adenoma Chest 2000 118 262 266 10.1378/chest.118.1.262 10893394

[B6] Rendleman N Quinn SF The answer is blowing in the wind: a pedunculated tumour with saw tooth flow-volume loop J Laryngol Otol 1998 112 973 975 10211226 10.1017/s0022215100142239

[B7] Verbraecken JA De Backer WA Upper airway mechanics Respiration 2009 78 121 133 10.1159/000222508 19478479 PMC2790795

[B8] Garcia-Pachon E Tracheobronchomalacia: a cause of flow oscillations on the flow-volume loop Chest 2000 118 1519 10.1378/chest.118.5.1519 11083722

